# Student Engagement Predictions in an e-Learning System and Their Impact on Student Course Assessment Scores

**DOI:** 10.1155/2018/6347186

**Published:** 2018-10-02

**Authors:** Mushtaq Hussain, Wenhao Zhu, Wu Zhang, Syed Muhammad Raza Abidi

**Affiliations:** School of Computer Engineering and Science, Shanghai University, 99 Shangda Road, Baoshan District, Post Code 200444 Shanghai, China

## Abstract

Several challenges are associated with e-learning systems, the most significant of which is the lack of student motivation in various course activities and for various course materials. In this study, we used machine learning (ML) algorithms to identify low-engagement students in a social science course at the Open University (OU) to assess the effect of engagement on student performance. The input variables of the study included *highest education level*, *final results*, *score on the assessment*, and the number of clicks on virtual learning environment (VLE) activities, which included *dataplus*, *forumng*, *glossary*, *oucollaborate*, *oucontent*, *resources*, *subpages*, *homepage*, *and URL* during the first course assessment. The output variable was the student level of engagement in the various activities. To predict low-engagement students, we applied several ML algorithms to the dataset. Using these algorithms, trained models were first obtained; then, the accuracy and kappa values of the models were compared. The results demonstrated that the J48, decision tree, JRIP, and gradient-boosted classifiers exhibited better performance in terms of the accuracy, kappa value, and recall compared to the other tested models. Based on these findings, we developed a dashboard to facilitate instructor at the OU. These models can easily be incorporated into VLE systems to help instructors evaluate student engagement during VLE courses with regard to different activities and materials and to provide additional interventions for students in advance of their final exam. Furthermore, this study examined the relationship between student engagement and the course assessment score.

## 1. Introduction

Web-based learning has become commonplace in education and can take many forms, from massive open online courses (MOOCs) to virtual learning environment (VLE) and learning management system (LMS). In MOOCs, students can study anytime and from nearly any location [1]. MOOCs provide a new way to train students, change the traditional approach to studying, and attract students from around the world. The best-known platforms are Coursera, Edx, and Harvard. Additionally, MOOCs have contributed to higher education [2]. In MOOCs and other web-based systems, students often register to download videos and materials but do not complete the entire course. As a result, the total number of activities a student engages in falls below the recommended threshold [3]. Therefore, teachers must understand the engagement of their students.

In the traditional approach to education, teachers take various steps to appraise students' levels of performance, motivation, and engagement [4], such as conducting exams, checking student attendance, and monitoring studying via security cameras. However, in web-based platforms, there are no face-to-face meetings, and it is difficult to determine student engagement levels in online activities such as participating in discussion forums or watching videos. Therefore, in web-based systems, student data represent the only source through which instructors can assess student performance and engagement.

Due to the absence of face-to-face meetings, web-based systems face some challenges that need to be addressed. The first and most important is course drop out. In web-based systems, dropping out is the principal problem that research has attempted to solve. In web-based systems, 78% of students fail to complete their courses [5]. The main reason students drop an MOOC course is the lack of student engagement, and the second most common reason is their inability to locate the requisite activities and materials for the next assessment [6].

An important element in reducing student dropout rates in a virtual learning environment (VLE) is to understand the engagement of students in meaningful activities. As student participation in course activities increases, the experiences become more engaging, and the probability of a student achieving a high assessment score and completing the e-learning course increases [7, 8].

### 1.1. Virtual Learning Environment (VLE)

In the current study, we used data from the VLE of Open University (OU) to investigate student engagement. The VLE stores course lectures, materials, and assessment information [9, 10]. The OU is the largest university in Europe. Nearly 200,000 students are enrolled in different courses in the OU [11]. Students enroll in a course through the VLE, and the VLE delivers different lectures, assignments, and materials from the OU to the students. One advantage of the VLE is that it allows an instructor to see the activities in which their students participate in the VLE and helps the instructor analyze those activities to understand student behavior [12]. The students interact with the VLE to watch lectures, complete assignments, and read materials. Finally, student interactions with the VLE are recorded and stored in log files. The logs contain student behavioral data, such as their interactions with the VLE system. An instructor can utilize these data to understand student behavior and mitigate the student dropout rate [13].

The students of the OU are generally divided into groups, and an instructor is assigned to each group. The instructor can guide these student groups through courses, for example, by answering their questions and grading their assignments. Additionally, the OU can use various types of intervention to support and motivate weaker students, e.g., through e-mail and face-to-face meetings [11]. However, the sheer number of students in the OU makes it increasingly difficult for the university to engage students in its courses via face-to-face meetings. Moreover, the number of instructors is limited, and it is not possible to contact all students in all courses. Therefore, an intelligent data system that predicts student engagement by analyzing logged student data is needed.

### 1.2. Significance of Predicting Student Engagement

Student engagement is the effort that a student spends on learning processes for the content of a specific course [14]. The most recent definition of behavioral engagement involves students who take part in discussion forums and show interest in MOOC materials [7]. Student engagement is an important research topic because a lack of student engagement affects the student's final grade, retention of material, and the course dropout rate [15]. A student who engages more in discussion forums and other MOOC activities usually does not drop out [16].

In web-based learning systems, a student's degree of engagement in educational learning is lower than that in traditional education systems [17]. Access to online VLE activities is used as a measurement of student engagement. Because the course involves web-based learning, often, no face-to face interaction occurs between students and the instructor. In web-based systems, it is difficult to measure a student's engagement using traditional methodologies (e.g., metrics such as class attendance, participation in discussions, and grades) [18, 19], because many of these predictors are not directly available in e-learning systems. Therefore, investigating students' engagement in web-based learning is a challenging task [20].

To accomplish our goals, we developed a predictive analytic model utilizing machine learning (ML) algorithms. The most appropriate ML predictive model was selected for analyzing student interactions in VLE learning activities and determining students' levels of engagement in VLE courses given that a lack of student engagement results in a high dropout rate [15].

Predictive models are currently used in many educational institutions [21]. A predictive model can help instructors guide students in succeeding in a course, and be used to determine which activities and materials are more important to the course assessment. Such models also enable instructors to engage students in different activities through the VLE, thereby encouraging the students to participate in the VLE course. Instructors must invest time discerning why student engagement in particular course activities and material is attenuated.

Our models can easily be integrated into VLE systems and can enable teachers to identify low-engagement students through different assessments, the use of different course materials, and the number of times VLE activities (e.g., *dataplus*, *forumng*, *glossary*, *resources*, *URL*, *homepage*, *oucollaborate,* and *subpages*) are accessed. Teachers can also spend more time on assessments and materials that are difficult for a particular group of students, enabling them to discover why an assessment is easy or difficult and providing supplementary intervention to students who need it.

A predictive system enables an instructor to automatically identify low-engagement students during a course based on activities from that online course. Given such detection, the instructor can then motivate (e.g., send an e-mail reminder) or identify difficulties during the course [22]. When a student receives an advisory e-mail from an instructor (i.e., an e-mail asking about any difficulty), on a weekly basis, the student is more likely to work hard and increase their engagement. Such communication is important because it assesses student workloads and addresses issues at an early stage of the course [23]. Apt advice will also improve student retention and decrease the course dropout rate.

Acquiring feedback is a challenge for instructors in an e-learning system after redesigning a course and related materials. The instructor can more effectively redesign a course and student materials using a predictive model of the progress of student and the finding can be used to improve the course and materials and increased engagement levels of students. Furthermore, teachers receive feedback on the courses they teach via e-learning systems and feedback focuses on the difficulty level, burden, and illustrative richness.

Tracking student engagement in different educational learning activities encourages high-quality learning, and comprehensive analysis of student engagement can help to minimize course dropout rates.

### 1.3. ML Techniques Used in a Predictive Model

We applied several ML algorithms as analytical learning approaches intended to predict student engagement during a VLE course and compared the resulting performance. ML is a field of artificial intelligence. ML algorithms can automatically find complex patterns from features extracted from existing data, enabling them to make smart decisions about current data [24].

The main tasks of learning analytics in education are to collect data, analyze these data and provide appropriate suggestions and feedback to students to improve their learning [25, 26]. With the help of predictive analytics, an instructor can also discover what students are doing with the learning material and how a student's assessment scores are related to that student's engagement level [27].

The cognitive ability of computers in some fields is still below that of humans, but due to ML algorithms, computer abilities are increasing quickly in domains such as e-learning, recommendation, pattern recognition, image processing, medical diagnosis, and many others. ML algorithms are trained using sample data as inputs and then tested with new data [28].

Instructors can use ML algorithms to obtain student-related information in real time, which helps them intervene during early course stages [29, 30]. ML is often used to build predictive models from student data; ML techniques can address both numerical and categorical predictor variables. Decision trees (DTs) are often used to construct trees and find predictive rules based on available data [31].

### 1.4. Innovation of the Current Study

In web-based systems, it is difficult for an instructor to determine the engagement levels of individual students because the students are not physically present [32]. The selection of an appropriate classification algorithm for student engagement prediction can be difficult because the recall and accuracy of these algorithms depend on choices made concerning their parameters, features, and study domains. In MOOCs, every student interacts with the course materials and performs or participates in certain activities. These interactions are recorded in student logs. In this work, we used these interaction data to identify low-engagement students in a social science course by first developing ML predictive models and then identifying the most appropriate model. The resulting predictive model can help instructors identify low-engagement students, receive feedback on VLE courses, and provide feedback to low-engagement students during early course stages. Further goals involved investigating which activities are the most appropriate and most affect student engagement and how student engagement is associated with assessment scores.

We used six types of ML classifiers (decision trees, JRIP, J48, gradient-boosted trees (GBT), classification and regression tree (CART), and a Naive Bayes classifier (NBC)) to build predictive (learning analytic) models that predict student engagement in different courses. These classifiers were selected because they accept both numeric and categorical attributes as inputs. The algorithms perform well on noisy data and are unaffected by nonlinear relationships between variables. They are white boxes in nature, and their rules can be easily interpreted and explained to users. Furthermore, using these algorithms, we can easily find the important variables in dataset.

In the current study, we used behavioral features (student features related to interaction with the VLE) to predict low-engagement students in an e-learning system. These features are readily available in almost every web-based system. Additionally, these features predict student engagement in a manner closer to that for a real world task (a traditional learning environment) [33].

The classifier inputs consisted of student e-learning activity data from the logs of a VLE system. After examining these data, we concluded the following: (1) the J48, DT, JRIP, and GBT classifiers were appropriate for predicting low-engagement students in the VLE course; (2) the number of student logins to *forumng* (discussion forums), *oucontent* (OU course materials), and *subpage* activities were strongly related to student engagement in the VLE system; and (3) highly engaged students achieved better results on course assessments than did low-engagement students. Furthermore, the results also indicated that the students who had lower-engagement in courses, achieved lower scores, and participated in fewer course activities.

This study considered the following research questions.

*Question 1*: Can we model the student engagement in different course activities by utilizing ML algorithms, and if so, which ML classifier offers optimal performance in predicting student engagement in the VLE course?

*Question 2*: Is it possible to identify the activities and conditions that are most important in web-based learning for predicting student engagement?

*Question 3*: How is a student's engagement in different VLE activities associated with that student's final score on an assessment?

The problem is described in Section 2. Related work is discussed in Section 3, Details about the materials and methods are presented in Section 4.  Section 5 describes and discusses the experimental results.  Section 6 provides conclusions and outlines future work.

## 2. Problem Description and Formulation

The OU is the largest university in the United Kingdom; it delivers more than a thousand online courses and offers online degrees [34]. The courses (modules) delivered by the OU are divided into 40-week intervals. Each course's content is divided into blocks. A different topic is taught in each course block, for example, Block 1, Part 1 is called Block 1, Week 1. Similarly, Block 1, Part 2 is called Block 1, Week 2. According to the course study plan, the study of a block occurs within a specific time.

The VLE contains the study material for each course, and each student's clicks per day are recorded in the VLE logs. The study material in the VLE is delivered via hypertext markup language (HTML), PDFs, and lecture format. The OU records activity and demographic data when a student uses the OU VLE system. The activity variables capture the type of communication through which the student is engaged with the VLE, and the activity types include *dataplus*, *forumng*, *glossary*, *oucollaborate*, *oucontent*, *resource*, subpage, *homepage*, and *URL*. The demographic data include the student's performance records. The instructor can use these data to monitor the student's engagement in different VLE activities.

In web-based systems, each group of students is supported by a specific instructor who can guide the students and provide feedback throughout the course. However, the resources for teacher-student interactions in the VLE are limited. As the number of students increases, it becomes more difficult for the OU staff to provide real-time support to all students.

The problem addressed in this paper involves reducing the dropout rate of students by identifying low-engagement students in the first course assessment stage, based on where students invested their time differentially and the activities they engaged in while completing the course assessments.

(1) $$\begin{array}{c} S={\left\{x_{i},y_{i}\right\}}_{i=1}^{N}. \end{array}$$

In the equation above, *S* is the training set in the study and *x*_*i*_ is an *N*-dimensional input vector that contains the input features. These features include the number of clicks on the VLE activities up to the student's completion of the first course assessment. *N* represents the number of students in the first assessment (*N*=383); *y*_*i*_ is the vector of the target class that determines the class of the input features *x*_*i*_, and *y*_*i*_ ∈ [1,0]. The result is assumed to be an indicator of engagement. When a student's level of engagement in the course is high through the first assessment, *y*_*i*_ is set to 1, and if the student's engagement level is low through the first assessment, then *y*_*i*_ is set to 0 (see Materials and Methods for the definition of engagement). The proposed functions to classify student engagement are as follows:

(2) $$\begin{array}{c} Y=f\left(S\right). \end{array}$$

Let *L* be a classifier. We trained each classifier *L* on *S* features. The *S* training set used to train each classifier *L* was a dyad of (*x*_*i*_, *y*_*i*_), where *x*_*i*_ denotes the historical record of the features and *y*_*i*_ is the class of feature *x*_*i*_ [35]. After training, we tested the classifiers using the test dataset, and the results are shown in Section 5.

Our results indicate that the VLE activity-type data are important for predicting student engagement during the assessment and that better-performing students have higher engagement through the first assessment. Additionally, previous studies also indicated that students who are less engaged have a greater chance of dropping out of their courses or failing subsequent assessments [36].

## 3. Related Work

Considerable research has been conducted to investigate student engagement in web-based learning systems and traditional educational systems. Such research has used different techniques and input features to investigate the relationship between student data and student engagement. For example, Guo et al. [37] studied student engagement while students watched videos. This study's input features were based on the time spent watching the video and the number of times the student responded to assessments. The study concluded that short videos engaged students to a greater degree than did prerecorded lectures. Bonafini et al. [38] used qualitative analysis and a statistical model (stepwise binomial logistic regression) to investigate student engagement in an MOOC discussion forum and while watching videos and related this engagement to student achievement. They used the number of posts submitted to a discussion forum, the number of videos watched, and postcontent review to study student engagement. The results indicated that the number of posts submitted in a discussion forum and the number of videos watched during a course were positively related to student achievement in the MOOC. Ramesh et al. [39] studied the engagement of MOOC students using a probabilistic model called probabilistic soft logic based on student behavior. Ramesh et al. [40] predicted student engagement/disengagement using student posts in a discussion forum. Beer [18] applied statistical methods to predict student engagement in a web-based learning environment and concluded that variables such as course design, teacher participation, class size, student gender, and student age need to be controlled for when assessing student engagement. Manwaring et al. [41] conducted a study to understand student engagement in higher education blended-learning classrooms. The study used a cross-lagged modeling technique and found that course design and student perception variables greatly affected student engagement in the course. Mutahi et al. [4] conducted a study to investigate the relationship between a student's final score and the student's engagement in material using a statistical technique and found that students who had high levels of engagement for quizzes and materials earned higher grades on the final exam. Aguiar et al. [42] developed an early-warning system using engagement-related input features and found that these variables are highly predictive of student-retention problems. Thomas and Jayagopi [43] measured student engagement using an ML algorithm based on students' facial expressions, head poses, and eye gazes. Their results showed that ML algorithms performed well at predicting student engagement in class. Atherton et al. [44] found a correlation between the use of course materials and student scores; students who accessed course content more often achieved better results on their exams and assessments. Bosch [45] studied the automatic detection of student cognitive engagement using a face-based approach.

Some previous studies have also investigated student engagement using log data [46, 47]. In recent years, researchers have investigated the effects of academic self-efficacy, teaching presence, and perceived ease of use on student engagement in MOOCs using statistical techniques [7]. Ding et al. [48] studied the effect of gamification on student engagement in online discussion forums. Wells et al. [49] studied student engagement and performance data using the LMS platform and concluded that student engagement increased as the exam approached. Additionally, they found a positive correlation between student performance and student engagement. Pardo et al. [50] revealed that student interactions with online learning activities have a significant impact on student exam scores. Other studies have found that student engagement is only weakly correlated with student performance in online knowledge surveys [51]. Hamid et al. [52] measured student engagement using an ML approach and concluded that the support vector machine (SVM) and the K-nearest neighbor (K-NN) classifiers are appropriate for predicting student engagement. Bote-Lorenzo and Gomez-Sanchez [53] predicted decreases in student engagement in an MOOC using an ML approach. Holmes [9] found that continuous assessment increased student engagement.

Several studies have shown that course outcomes are positively correlated with student engagement [54, 55]. For example, Atherton et al. [44] showed that students who access web-based system study materials daily and undergo regular assessments achieve high exam scores. Other research results show that high-engagement students tend to earn good grades on course quizzes and assessments [4]. Rodgers [56] found that student interactions with an e-learning system were significantly correlated with course outcomes.

However, most of the previous work on engagement has focused on traditional education in universities and schools but has neglected student engagement in web-based systems. Additionally, the previous work related to student engagement has been based on statistical analysis, survey, and qualitative methods; however, these statistical approaches cannot reveal the hidden knowledge in student data. Moreover, statistics-based and qualitative methods are not easily generalized, nor are they scalable. Surveys are not a good option for measuring student engagement; for example, younger children cannot understand the questions, and completing the surveys requires a large amount of time. Another downside of these studies is that they are based on student behaviors and emotions, as well as the course design; however, student engagement can also depend on student participation in learning activities.

The current study uses ML techniques to predict low-engagement students in a web-based learning system from VLE log data. Accessing the log data using ML techniques does not interrupt students [33], and the data are not time dependent. However, to the best of our knowledge, no research that predicts student engagement in a web-based system using ML techniques and then compares those predictions with student assessment scores has previously been conducted.

## 4. Materials and Methods

**Figure 1:**
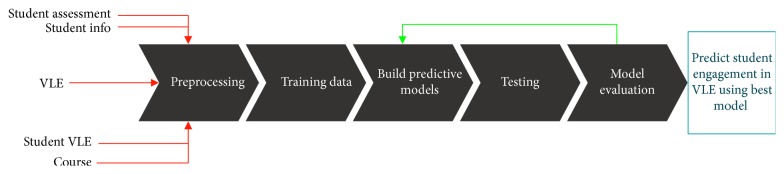
Flow of the student engagement prediction process.

In this study, we utilized various ML techniques to study student engagement in different VLE activities. The selected techniques were suitable for both domain and categorical educational attributes. The main steps in the current study are presented in Figure 1. Brief details of the ML, training, testing, and input data are provided below.

### 4.1. Machine Learning Technique

Various types of ML techniques have been used as predictive models. The ML techniques tested as predictive models in the current study are described below.

#### 4.1.1. Decision Tree (DT)

A DT has a tree-like structure with internal nodes represented by rectangles and leaves represented by ovals. An internal node has two or more child nodes. The internal nodes represent dataset features, and the branches represent the values of these features. Each leaf contains a class related to the dataset [57].

The DT is trained with a training set containing tuples. Finally, the DT is used to classify a dataset with unknown class labels [57]. DTs are primarily used to process information for decision-making [58].

The tree is constructed from the dataset by determining which attributes best split the input features at the child nodes. In this case, we used the concept of information gain which is dependent on information theory. When a node has minimum entropy (highest information gain), that node is used as a split node [59]. A DT is important when a study seeks to determine which features are important in a student prediction model [60]. The rules for DTs are easy to understand and interpret, and we know exactly which classifier leads to a decision.

#### 4.1.2. J48

A J48 decision tree belongs to the DT family; it both produces rules and creates the tree from a dataset. The J48 algorithm is an improved version of the C4.5 algorithm [61]. It is a sample-predictive ML model that predicts the target values of an unseen database based on the different values of input features in the current dataset. The rules of this approach are easily interpreted. Moreover, this method is an implementation of the ID3 (interactive dichotomize) algorithm and is a supervised ML algorithm used primarily for classification problems. The internal nodes of a J48 decision tree represent the input features (attributes), and the branches of the tree represent the possible values of the input features in the new dataset. Finally, the terminal nodes (leaves) display the final values of target variables [62]. The attribute-selection process is based on the information gain method (gain ratio) [63]. The J48 decision tree works for both numeric and categorical variables; moreover, it determines the variables that are best at splitting the dataset [30]. The attribute with the highest gain ratio reflects the best split point.

#### 4.1.3. Classification and Regression Tree (CART)

A CART works in the same way as ID3 but uses the Gini index method for feature selection. The variable with the smallest Gini index value is considered the split variable [59]. When the target variable of a training dataset is nominal, CART constructs a classification tree, whereas when the target value is numeric, CART constructs a regression tree [57]. A CART is simple to understand and visualize. A CART is mostly used for variable screening and feature selection and can handle both numeric and categorical data. A CART checks all possible divisions for all the predictors. When the optimal division is found, the CART again performs the search process involving every CART node until further division is not possible [63].

#### 4.1.4. JRIP Decision Rules

This technique is a popular classification algorithm that uses repeated incremental pruning to produce error reduction (RIPPER) to create rules from a dataset. JRIP uses the minimum error attribute of class prediction. Moreover, JRIP uses an association rule with reduced error pruning [64]. The partial decision tree (PART) algorithm creates a DT and rules from the dataset [65]. Then JRIP generates a confidence score, which depends on the allocation of a training example classified by the JRIP rules [66]. For example, if we wanted to classify 10 training samples using the JRIP rules [66], the allocations of those samples could be made such that 7 are positive and 3 are negative. In this case, the confidence assigned to each predictor would be 0.7 [66].

#### 4.1.5. Gradient Boosting Trees (GBT)

The GBT algorithm is used for solving supervised learning problems and can be applied for both classification and regression, as well as for ranking problems. Usually, in supervised problems, the training data (which can include multiple features) are used to forecast the target class. The GBT algorithm is popular and has been integrated into both educational and commercial applications. The GBT is constructed greedily. The construction process starts from the root node, and leaf splits are based on the mean square error.

#### 4.1.6. Naive Bayes Classifier (NBC)

The NBC is based on Bayes' theorem and performs well for data with high input dimensions. The NBC works on independent features in datasets and requires less computational time and less training data than do other ML algorithms [67].

### 4.2. Data Description

The present study examined data from a module (lesson) of a social science course attended by OU students working via the VLE system that addressed a particular topic in a given course block [68]. The VLE system provides different course topics in different course blocks. This VLE delivers various courses to students, and students can enroll in the courses from different locations [6].

The number of students enrolled in the social science course for the July 2013 session was 384. We used only the July 2013 student records (384 students) that applied to the period through the first assessment from the social science course data. Based on the first assessment scores, the instructor can determine the low-engagement students at an early point in the course. We extracted three types of data: demographic (*highest education level*), performance (*final results* and *score on the assessment*), and learning behavior (*number of clicks on VLE activities*) data. The behavioral data included the number of clicks on activity types such as *dataplus*, *forumng*, *glossary*, oucollaborate, *oucontent*, *resources*, *subpage*, *homepage*, and *URL* as well as the number of times each student accessed VLE activities through the first course assessment.

**Figure 2:**
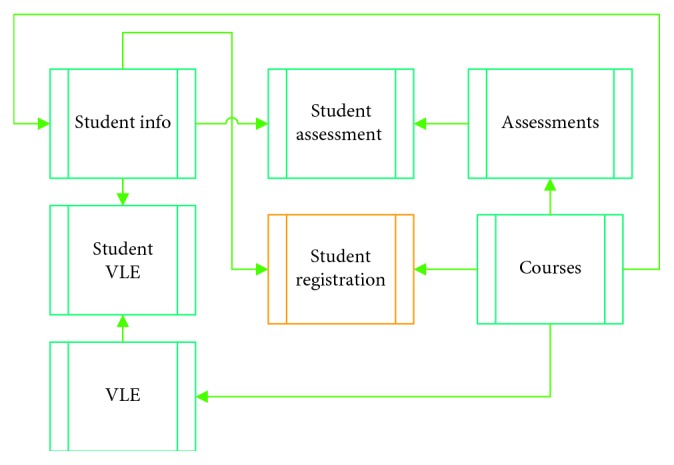
Details of the tables included in the OU dataset.

One problem is that the selected attributes are stored in different tables (student info, student assessment, assessments, student VLE, courses, and VLE) in the OU data, as shown in Figure 2. The student info table contains the students' demographic information and the results of each course [68]. The course table contains information about the courses in which students are enrolled [68]. The registration table contains student record timestamps and course enrollment dates [68]. Assessment information is recorded in the assessment table [68]. The student-assessment table contains the assessment results for different students [68]. The interaction information of different students regarding different materials and activities is stored in the student-VLE and VLE tables [68]. The VLE interaction data consist of the numbers of clicks students made while studying the course material in the VLE. Each course activity is identified with a label, for example, *dataplus*, *forumng*, *oucontent*, etc.

### 4.3. Preprocessing

The data provided by the OU could not be directly used as inputs in the ML model. Initially, the input features of the current study were sourced from different tables (as discussed above) and needed to be integrated into a single table. We performed various preprocessing steps on the data using MATLAB to format the raw data into a form acceptable for ML algorithms.  Algorithm 1 shows the feature extraction steps. The preprocessing steps are listed below.

#### 4.3.1. Feature Extraction

We transformed the open dataset of the VLE by extracting a set of student features related to their performance and interaction activities because these features were strongly related to student engagement in the course. We used MATLAB to calculate the total number of times a student logged in to any VLE activity.

We transferred the data into an ML-compatible layout in which each row index was a student ID and each column index was a student's feature. Thus, each attribute was related to the first assessment in the social science course.

#### 4.3.2. Label (High and Low Engagement) Extraction

Engagement identification is essential in web-based education because it affects student performance, the student dropout rate, and retention in e-learning courses. The most highly engaged students tended to obtain high scores.

**Figure 3:**
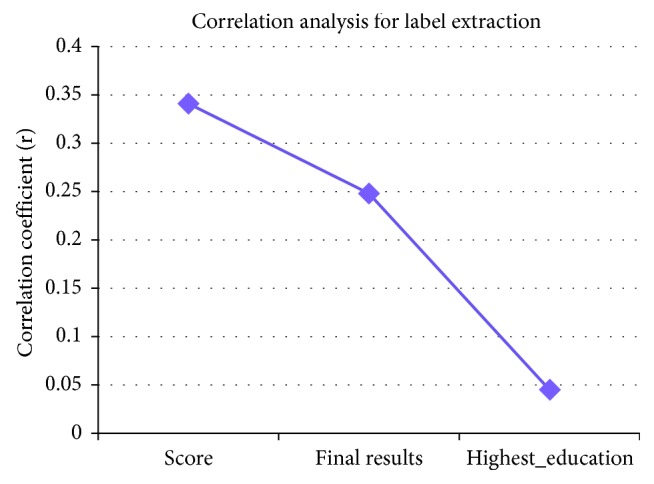
The correlation coefficient between the dependent variable *(total number of clicks on VLE activities)* and independent variables *(score on the assessment, final results, and education level)* of the students. *Note*: *score on the assessment* (score); *highest education level* (highest_education)

Before developing the predictive models, we established a label or definition of engagement; thus, the total number of times a student accessed the VLE activities (*total number of clicks on VLE activities*) was assumed to be an indicator of engagement. The detection of student engagement merely by observing student behavior is challenging [69] because students can sometimes appear to be busy but fail to complete any learning tasks. Moreover, the prediction of student engagement in VLE courses by simply counting clicks during VLE activities is difficult because students sometimes click on other, unimportant activities, such as Facebook and Twitter. Additionally, in some cases, students spend little time in the VLE but achieve a high score on the course assessment. Therefore, the total number of clicks on the VLE is insufficient for measuring student engagement in the VLE course [9]. Instead, the criterion for measuring engagement in the current study was jointly based on four variables: the first assessment score (*score on the assessment*), the student education degree before registering for the course (*highest education level*),the final exam result after completing the course (*final results*), and the *total number of clicks on VLE activities*. To discern the impacts of these four variables on student engagement, we conducted statistical analyses (Spearman's correlation) using SPSS to assess the relationship among the *total number of clicks on VLE activities* and the *highest education level, score on the assessment,* and *final results* in the course for each student, based on a significance level of 0.05. The results are shown in Figure 3.

Figure 3 illustrates that two variables (*final results* and *score on the assessment*) were significantly correlated with the dependent variable (*total number of clicks on VLE activities*). Therefore, we define engagement through three variables (*final results, score on the assessment,* and *total number of clicks on VLE activities*). The details of the above variables are given later in the paper (see the section “Predictors that affect student engagement in the Web-based system”).

Figure 3 shows that student engagement is related to the *final results*, *score on the assessment* and *total number of clicks on VLE activities* for each student, based on the large r-values of these variables. The most highly engaged students achieved higher scores and better results on the exam. The *highest education level* variable was omitted because it had a low *r* value that was statistically nonsignificant. Therefore, we define engagement as follows:

(3) $$\begin{array}{c} Z=O\left(\text{Exce},\text{Que},\text{Act}\right), \end{array}$$

(4) $$\begin{array}{c} O_{\sigma \left(\text{Exce},\text{Que},\text{Act}\right)}=\left\{\begin{array}{cc} 1, & Z=\text{Exce }\vee \left(\text{Qua }\wedge \text{ Act}\right)⟶\text{High engagement},\\ 0, & \text{Otherwise}⟶\text{Low engagement}, \end{array}\right. \end{array}$$

where *Z*_*i*_ represents the student's engagement level (high or low) on the assessment and *Z*_*i*_ ∈ {1,0}.The “OR” operator is denoted by ∨ and “AND” operator is denoted by ∧.

Additionally, Exce denotes students who achieved excellent scores on the first assessment (*score on the assessment* ≥ 90%), Qua denotes those students who are qualified (*final results* = Pass) and Act represents those students who are active during the course (*total number of clicks on VLE activities* ≥ average clicks of students). After establishing the engagement label, all the training data were labeled using the engagement rules presented above.

Student engagement can be measured using different methods, such as questionnaires, external observers, and physiological measures; however, in these methods, students can disturb, and these methods are not scalable [43]. Furthermore, according to prior research, measuring student engagement using total clicks during course does not guarantee that students are highly engaged [9]; therefore, we measured student engagement based on excellent (Exce), qualified (Qua), and activate (Act) during the course. The results conclude that high-engagement students can achieve high scores on assessments (excellent) and pass (qualified) final exams and are more active during the VLE course (active).

#### 4.3.3. Feature Selection

In this study, we predicted low-engagement students using the number of clicks on the VLE activities; therefore, we consider only the activities-related features (i.e., number of logins to *dataplus*, *forumng*, *glossary*, *oucollaborate*, *oucontent*, *resources*, *subpage*, *homepage, and URL*). Learning is a process that occurs when students interact with course materials and receive instruction [70]. These features describe how students participate while taking the VLE course.

#### 4.3.4. Missing Values

Some values were missing from the dataset. We substituted zero values for these missing data and interpreted the zeros as indicating that the student did not login to those activities through the first assessment.

### 4.4. Predictors that Affect Student Engagement in Web-Based Systems

In web-based systems, the most important predictors are the student activities and the materials used before the course assessment. Further details on the predictors used in the current study are given below.

#### 4.4.1. Activity Type

Students engaged in a range of VLE activities, namely, *forumng*, *dataplus*, *glossary*, *oucollaborate*, *oucontent*, *resources*, *subpage*, *homepage,* and *URL*, while completing the course assessment. These activities provide important information for predictive analysis. The number of times each student clicks on each of the activities is recorded daily in a time-stamped log file that indicates the time the student spent on each activity. The *forumng* variable references the discussion forum, where students can discuss problems with each other. The forum is also a space where students can submit questions to better understand the subject [60]. *Resources* consist of lecture notes, books, lecture slides, and other course materials in HTML and PDF formats [60]. The *oucontent* variable contains study materials in HTML format related to the specific course studied. The *subpage* variable reveals the student's navigation path through the VLE structure [60]. The *homepage* variable reflects the first screen of every course; these screens are visited by a student before accessing other course material. The *glossary* includes details about the OU and higher education acronyms. The *dataplus* variable references a module developed by the OU that allows students to see their own records that have been stored in the database. Additionally, with the help of this module, producing a SQLite3 database is both customizable and portable for web-based systems. Furthermore, students can easily export the OU database.

#### 4.4.2. Student ID

This variable is a unique identification number for a student in the OU records.

#### 4.4.3. Highest Education Level

This variable reflects the highest degree a student achieved before registering for the course. Student educational level is also correlated with the degree of student engagement in the web-based system.

#### 4.4.4. Total Number of Clicks on VLE Activities

This variable represents the total number of times a student accessed VLE activities up to the assessment. Decreases in this variable are a warning sign of low-engagement. The total number of times VLE activities were accessed was considered an indicator of student engagement and is an important predictor for student engagement and student participation [18, 71].

#### 4.4.5. Score on the Assessment

This attribute represents the score obtained by a student after completing the first assessment in the social science course.

#### 4.4.6. Final Results

This variable represents the student's final exam results after completing the course, and the possible values are pass or fail. This important variable reflects student effort in the course.

All the aforementioned variables are associated with student engagement at the OU. However, learning is a complex process that is also affected by other factors, such as teacher participation in discussion forums, course design, class size, teacher's experience, teacher's conception of learning, teaching styles, and other factors [18, 72].

### 4.5. Building and Testing the Predictive Model

After preparing the data, we trained models using the student training data. In this step, the decision tree algorithms DT, JRIP, J48, GBT, and CART and the NBC algorithm were used.

We constructed an Excel file from the training data and uploaded it to Rapid Miner. Rapid Miner includes the entire visualization module and predictive module of the decision tree. Therefore, we could easily construct the decision tree algorithms and the NBC from these data.

The current study classifiers were trained using a dataset constructed from the VLE data. The input features used in training were the total number of clicks on the VLE activities completed in the VLE course, and the target variable was the predicted student level of engagement (high or low) throughout the OU course.

In the training phase, we supplied the inputs and the corresponding data classes to the ML classifier to allow the classifiers to discover the patterns between the input and output [73]. Finally, the trained models used these patterns to classify unseen data [73].

We used a 10-fold cross-validation method to train and test the current student models. Cross-validation is primarily utilized to assess model performance [74]. In k-fold cross-validation, the data are divided into k different subsets, the model is trained using k-1 subsets, and the remaining subset is used for testing. The average performance obtained from this method provides a good estimation of model performance. [74].

### 4.6. Performance Metrics

After training the classifiers in the current study, we assessed the performance of the learning models using previously unseen data. We obtained the prediction results for the models with the test data and counted the number of true positives, true negatives, false positives, and false negatives that were used to evaluate performance. Through this process, we obtained the numbers of true positives (low-engagement) and true negatives (high engagement), as well as the number of false positives and false negatives.

Our main goal in this study was to minimize the false-negatives rate (i.e., the number of low-engagement students incorrectly identified as high-engagement students). Therefore, we selected the model with the highest recall [75]. We used the following performance metrics to measure the quality of the ML model predictions.

#### 4.6.1. Accuracy

The first metric was accuracy, which is the number of low-engagement students correctly predicted as having low-engagement during the course [75, 76].

(5) $$\begin{array}{c} \text{Accuracy}=\frac{\text{true positive}+\text{true negative}}{\text{true positive}+\text{true negative}+\text{false positive}+\text{false negative}}. \end{array}$$

#### 4.6.2. Recall

Next, we calculated the recall, which indicates the fraction of all the students in the dataset who have low-engagement and who the classifier correctly recognized as having low engagement [75, 76]. An ML model with a high recall is considered to have satisfactory performance.

(6) $$\begin{array}{c} \text{Recall}=\frac{\text{true positive}}{\text{true positive}+\text{false negative}}. \end{array}$$

#### 4.6.3. Area under the Curve (AUC)

The receiver operating characteristic (ROC) curve is a popular method for assessing classifiers when a dataset is unbalanced [42]. The X-axis of an ROC represents the false-positive rate, and the Y-axis represents the true-positive rate [42]. The AUC ranges from a minimum of 0 to a maximum of 1. When the AUC of a model is greater than 0.5, it is considered a good model [22].

#### 4.6.4. Kappa

When a model has a kappa value of zero, its performance is poor; in contrast, a value near 1 indicates that the model has achieves good performance [30].

## 5. Experiments and Results

In this part of the study, we predicted the numbers of low-engagement students from the different activities of a VLE course using features related to student activity. To answer the research questions of the current study, we performed several experiments. We used the ML algorithms and the Rapid Miner tool to build the learning models, as described below.

### 5.1. Data Visualization and Statistical Analysis of the Data

To understand the student data, we performed statistical analyses of the number of times students clicked on activities and the student engagement level. We also visualized the dataset. This step is important in ML studies [76] because the performance of a predictive model sometimes decreases when the data quality is poor [77].

We visualized the input variables (student clicks on VLE activities) of the OU course to illustrate how important the input variables are in predicting low-engagement students at the first assessment point of a social science course. The results were used to better understand the student data [26].

**Figure 4:**
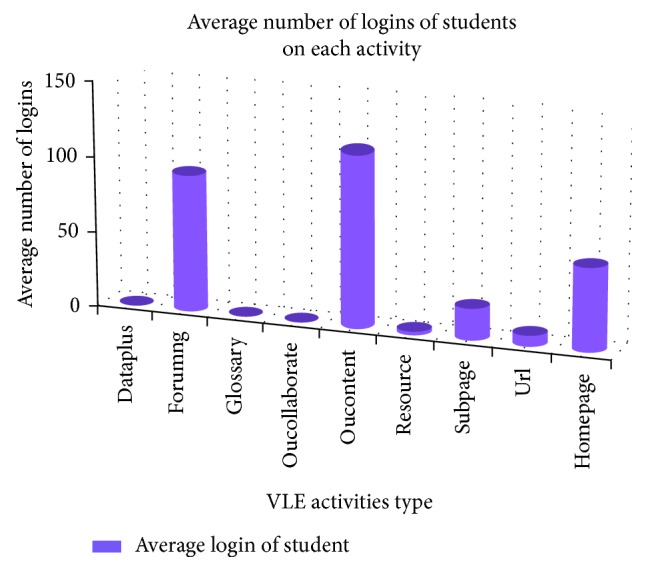
Average number of student logins for each course activity.

We visualized the number of clicks per activity, for example, *forumng*, *oucontent*, *homepage,* etc.  Figure 4 presents the number of clicks per activity, which indicates how much time the students spent on each activity.

Figure 4 shows that the number of logins a student has for *forumng* and *oucontent* activities is greater than those for other activities. Additionally, student *forumng* and *oucontent* engagement is greater when completing the first assessment. These findings demonstrate that *forumng* and *oucontent* have high importance in predicting low-engagement students.

In the second step, we determined how much the input features of the current study were correlated with the output. Therefore, before applying the ML algorithms, we conducted a statistical analysis (Spearman correlation) to determine the significance between the dependent variable of the study (level of engagement) and the independent variables (*score on the assessment* and the number of clicks on VLE activities, namely, *dataplus*, *forumng*, *glossary*, *oucollaborate*, *oucontent*, *resource*, *subpage*, *homepage*, and *URL*). A spearman correlation is appropriate for both continuous and discrete features [78].

After conducting the Spearman correlation analysis, each independent variable in the current study received a correlation coefficient (*r*) that reflected the strength and direction of the linear relationship between the tested pair of variables [79]. The results are shown in Table 1.

The statistical results show that student clicks on *forumng, oucontent, subpage,* and *URL* were moderately correlated with the level of engagement in VLE activities, whereas the number of student clicks on *resources* and *oucollaborate* were weakly correlated. Moreover, the number of clicks on the *homepage* was highly correlated with the level of engagement. Table 1 shows that the number of clicks on *glossary and dataplus* were unrelated to the student level of engagement in VLE activities [80].

Although some of the selected predictor variables were not significant, we included all the predictor variables in our experiment, following the advice of Luan and Zho [81]. According to Luan and Zho [81], nonsignificant variables can be important in some records [82].

Table 1 indicates that the seven independent variables, namely, the number of clicks on *forumng*, *oucontent*, *subpage*, *oucollaborate*, *resources*, *homepage,* and *URL were* significant (*P* values < 0.05) with respect to the dependent variables. These independent variables are meaningful and were used in subsequent experiments. However, this analytical statistic does not reveal the hidden information in the data [76].

Because most of the input variables in the current study were significant predictors of student engagement, there was room for further application of the ML algorithms.

### 5.2. Results and Discussion

At this point, we had sufficient knowledge of the data to build predictive models. The study was conducted to determine which learning algorithms are most suitable for predicting low-engagement students based on their activities related to features during a VLE course and to determine which activity types are important in predicting student engagement. A measure of engagement allows teachers to understand how a student is engaged with the learning content during a course. We performed several experiments to answer the following questions.

*Question 1*. Can we model the student engagement in different course activities by utilizing ML algorithms, and if so, which ML classifier offers optimal performance in predicting student engagement in the VLE course?

To explore this question, we determined the best ML algorithms to predict low-engagement students and performed the first experiment. In this experiment, the input features were the students' clicks on VLE activities (*dataplus*, *forumng*, *glossary*, *oucontent*, *resource*, *subpage*, *homepage, and URL*) in a VLE course, and the target variable was the students' level of engagement.

To estimate how the classifier could generalize the unseen data, we divided the data using a 10-fold cross-validation method. We followed this procedure to determine student engagement as predicted by each ML model.

In the first experiment, we used the DT, J48, JRIP, GBT, CART, and NBC algorithms to predict student engagement using the student interaction data. We used the Rapid Miner tool to build the ML models and determined the accuracy of each algorithm using 10-fold cross-validation.

The DT is a supervised ML algorithm that is simple and easy to understand. We used the following optimum parameters to train the DT: criterion = gain ratio, maximal depth = 20, confidence = 0.25, minimal gain = 0.1, and minimal leaf size = 2. Finally, we obtained an accuracy of 85.91% after applying 10-fold cross-validation.

Table 2 shows that the DT predictive model correctly classified 283 of 303 low-engagement students; therefore, its true-positive rate (sensitivity or recall) was 0.9340, with a false-positive rate of 0.425.

In the second phase of the first experiment, we used the J48 classifier and evaluated the related model via 10-fold cross-validation. We obtained the best performance results using the following parameters: confidence threshold (C) = 0.25 and minimum number of instances per leaf = 2.0.

Table 3 shows that the J48 classifier correctly classified 289 of 305 low-engagement students. The true-positive rate (sensitivity or recall) of this model was 0.947, and its false-positive rate was 0.358. Finally, the accuracy of the classifier was 88.52%. The results are shown in Table 3.

In the third phase of the first experiment, we built a JRIP decision tree model using Rapid Miner. The default parameters for the JRIP models during the training stage were set as follows: F (number of folds per REP) = 3.0, *N* (minimal weights of instances with a split) = 2; *O* (number of optimization runs) = 2.0, and S (seed value used for data randomization) = 1.0.

Table 4 shows that the JRIP decision tree correctly classified 227 of 243 low-engagement students. The true-positive rate of the JRIP model was 0.934, the false-positive rate was 0.342 and the accuracy was 83.27%.

In the fourth phase of the first experiment, we built a GBT model to predict low-engagement students in the VLE course. The default parameters of the GBT were as follows: number of trees = 20; maximal depth = 5; min rows = 10.0; and number of bins = 20. The GBT correctly classified 294 of 323 low-engagement students. The true-positive rate (recall or sensitivity) of the GBT was 0.910, and the false-positive rate was 0.383. Moreover, this model achieved high accuracy (86.43%) based on the default parameters. The results are shown in Table 5.

In the fifth phase of the first experiment, we developed a CART model using the OU student data.  Table 6 shows that the CART model correctly classified 235 of 263 low-engagement students. The default parameters used in this model were as follows: *S* (random number of seeds) = 1; *M* (the minimum number of instances at the terminal nodes) = 2.0; and *N* (number of folds used in the minimal cost-complexity pruning) = 5.0. The true-positive rate (recall or sensitivity) of the CART model was 0.893, the false-positive rate was 0.333, and the accuracy was 82.25%.

We applied the NB (kernel) classifier to our data to calculate the probabilities associated with highly engaged and lowly engaged students. We implemented the NBC using kernel density estimation and obtained good performance with the following parameters: estimation mode = greedy; minimum bandwidth = 0.1; and number of kernels = 10. The true-positive rate (recall and sensitivity) of the NBC was 0.900, the false-positive rate was 0.50, and the accuracy was 82.93%. The results are listed in Table 7.

**Figure 5:**
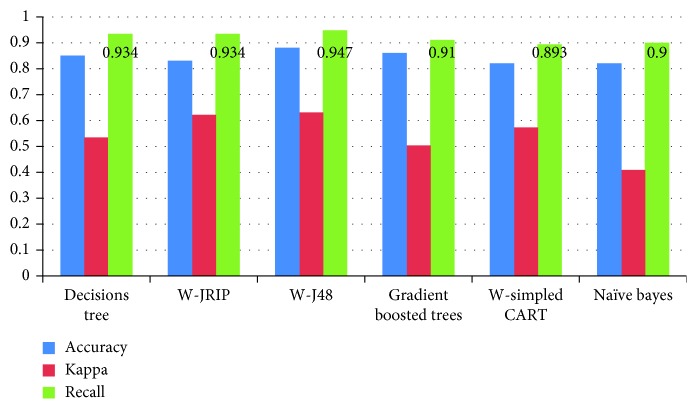
Visualization of the accuracy, kappa, and recall of the ML models used in the current study.

A comparison of the results of the six models (Table 8 and Figure 5) showed that J48, GBT, DT, and JRIP predicted low-engagement students with high accuracy (88.52%, 86.43%, 85.91%, and 83.27%, respectively) based on student clicks on different activities.

When data are unbalanced (i.e., when the number of records of one class is less than that of another), accuracy alone does not always indicate that a classifier has achieved good performance in predicting low-engagement students (the unbalanced problem) [43]; therefore, we checked the recall (sensitivity), ROC, and kappa values of the classifiers.

To identify low-engagement students in the OU course, recall is paramount, but if we want to identify high-engagement students (those with a larger number of clicks on activities), then accuracy is important. In this study, our goal was to identify low-engagement students; therefore, we focused on recall.

In our model, the recall results reflect how many of the low-engagement students were correctly identified as low-engagement out of the total number of low-engagement students in the dataset. Given such identification, teachers can give feedback and sent warning messages to those students who may need to work harder.

In the first experiment, the study found that the recall, kappa, and accuracy values of the J48, GBT, DT, and JRIP models were better than those of the NBC classifier and CART. Thus, J48, JRIP, GBT, and DT were selected as the appropriate classifiers for VLE data. DT achieved a recall for low-engagement student prediction of 0.934, a kappa value of 0.534, and an accuracy of 85.91%, and J48 achieved a recall of 0.947 for low-engagement students, a kappa value of 0.630, and an accuracy of 88.52%. The GBT had a recall of 0.910, a kappa of 0.503, and an accuracy of 86.43% in the current experiments. Finally, JRIP achieved a recall of 0.934, a kappa of 0.621, and an accuracy of 83.27%. These results indicate that the recall of J48 is slightly greater than that of the others models, which suggests that the performance of the J48 classifier in predicting low-engagement students is good compared to the alternatives.

**Figure 6:**
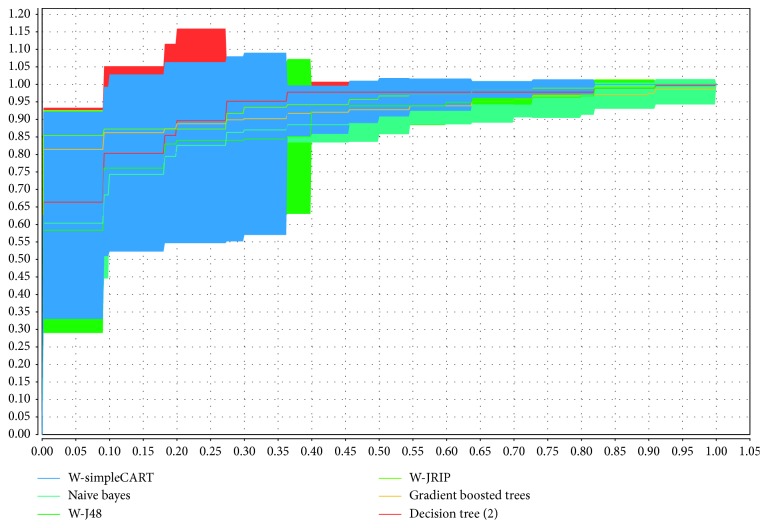
ROC curves of the models applied in this study.

In the first experiment, we also compared the performances of the learning algorithms based on the ROC curves. We computed the AUC value of each classifier, and they ranged from 0.8 to 0.5. The ROC curves of our models represent the probability that low-engagement students in the sample are correctly identified as low. A high AUC means that the classifier has good performance, and the AUC value is close to 1. A low AUC indicates that the classifier performs poorly.  Figure 6 shows that the J48, JRIP, GBT, and DT classifiers achieved better AUC values than did the other algorithms and thus performed better. The ROC curves of the other models indicate that these models achieved inferior performance for the studied dataset.

The experimental results show that J48, JRIP, GBT, and DT are appropriate algorithms for identifying low-engagement students in an OU course. When low-engagement students can be identified, teachers can utilize these models to alert low-engagement students in advance and learn more about the low-engagement students. The NBC and CART classifiers were less accurate than the GBT, J48, JRIP, and DT classifiers and did not perform as well in predicting low-engagement students during a VLE course. Figure 5 and Table 8 also show that low-engagement students can be predicted with reasonable accuracy and recall based on the number of clicks on VLE activities prior to the first assessment.

*Question 2.* Is it possible to identify the activities and conditions that are most important in web-based learning for predicting student engagement?

To understand which VLE activities are important for student engagement prediction, we explored the second question by building a decision tree using the DT classifier because the explanations generated through decision trees are easily generalized and understandable [83].

**Figure 7:**
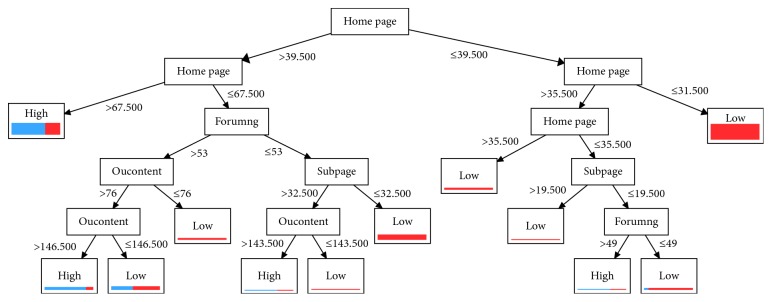
DT used to predict student engagement in VLE course.

We applied the DT to discover more details about the students.  Figure 7 shows the DTs constructed from the VLE dataset. Several interesting observations can be drawn from building the DT classifier.

First, the analysis demonstrates that student clicks on *homepage*, *forumng*, *oucontent*, and *subpage* are the most important predictors of student engagement in the VLE course because these features appear most frequently in the classification model. Other features, such as the number of student clicks on *dataplus*, *glossary*, *oucollaborate*, *resources,* and *URL* are not important predictors of student engagement through the first assessment and are not shown in Figure 7.

Second, from Figure 7, we conclude that *forumng* and *oucontent* are the most important types of activities for predicting low-engagement in the social science course. The number of messages posted and replied to in the discussion forum (*forumng*) may also be related to the engagement of students in the course. Additionally, instructor involvement in the discussion forum could further improve student engagement [18].

Third, Figure 7 further demonstrates that highly engaged students accessed more OU course-related content (*oucontent*) during the course and had a higher level of participation in the discussion forum; therefore, they tended to interact more with other students. In contrast, low-engagement students clicked less in discussion forums and on oucontent.

Fourth, Figure 7 shows that the *forumng*, *oucontent*, and *subpage* activities have a deleterious effect when a student has low-level engagement.  Figure 7 shows that when a student's participation in activities such as *forumng*, *subpage,* and *oucontent* is low, the student's engagement level will also be low.

The features selected by our models provide good information that instructors can use to provide confident interventions for their students before the end of a course. According to prior research, student retention can also be improved by improving the level of student interaction in the course [30, 84, 85]. The DT generated by the DT algorithm shows how the activities of an OU course can be used to predict student engagement, and these findings also reflect the confidence level of each student.

Furthermore, we constructed the following interesting rules from the JRIP tree.

1. (*homepage* ≥ 55) and (*oucontent* ≥ 171) and (*subpage* ≤ 42) and (*oucontent* ≥ 189) ≥ Label = High (30.0/2.0)
2. (*homepage* ≥ 68) ≥ Label = High (79.0./30.0)
3. (*forumng* ≥ 145) ≥ Label = High (15.0/3.0)
4. (*oucontent* ≥ 147) and (*foruming* ≥ 53) ≥ Label = High (15.0/3.0)
5. Label ≥ Low (244.0./7.0)

These rules show that student clicks on *homepage*, *oucontent,* and *forumng* are significantly related to student engagement. Moreover, the rules can be interpreted as follows.

When a student spends more time in the discussion forum (*forumng*), engagement in course activities is high. Additionally, high-assessment-score students spent more time creating new content and pages via which their assessments were submitted and used subject material more frequently to clarify concepts than did low-score students.

When the number of clicks of a student on a discussion forum *(forumng)* is ≥145 OR homepage (*homepage*) ≥68 OR *oucontent* ≥ 147, then the student's engagement level in the OU course is high.

Finally, experiments 1 and 2 showed that JRIP, J48, DT, and GBT are appropriate ML models for predicting low-engagement students in the VLE course. Additionally, the results showed that four features strongly affect student engagement and course outcomes, namely, the number of clicks on *forumng*, *oucontent*, *homepage,* and *subpage*. The rule sets selected through this analysis can help instructors better design their course content and identify low-engagement students at an early course stage. The performances of the GBT, J48, DT, and JRIP models, which were sufficient for practical applications, were good for the following reasons.

1. GBT, J48, DT, and JRIP can be effectively applied for both numerical and categorical attributes [86]. Additionally, these models are white box models, and their results are easily interpreted [87].
2. The performance of JRIP is good in the presence of noise [66], and it produces rules that can be easily understood by teachers.
3. DTs can handle more predictor variables than can logistic regression and other models [82], and they can find complex and nonlinear relations between dependent and independent variables [82].
4. J48 can handle missing values and unknown levels, and it provides good stability and easily understandable results [61]. J48 can also handle both discrete and continuous values [88].
5. DTs can produce rules that are easily handled and understood by humans. Additionally, DTs perform rapid classification of unknown data, without requiring complex computations [61].

*Question 3*: How is a student's engagement in different VLE activities associated with that student's final score on an assessment?

To address this question, we determine how student engagement is related to the first assessment scores by conducting a third experiment in which we analyze student data related to student engagement in VLE activities and the first assessment score in the VLE course. According to previous work, student engagement has a positive effect on grades, test scores, retention, and graduation [89].

The Spearman correlation (*r*) between student assessment scores and student engagement was 0.351 (Table 1), which indicates that engagement is positively correlated with assessment scores.

**Figure 8:**
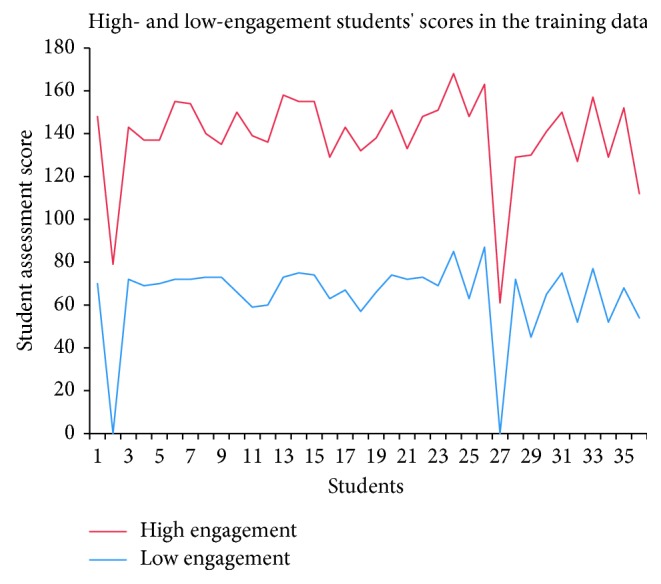
Student assessment scores compared to student engagement levels in the training data.

Figure 8 shows the relationship between student assessment scores and engagement level for the training data. Figure 8 further indicates that students with high assessment scores in the training data show high engagement. Therefore, we applied the J48 decision tree to study these relationships in the test data. We split the dataset into two portions, training and testing, with allocations of 75% and 25%, respectively. We trained the J48 classifier on training data and tested it on unseen data (testing data). In other words, we compared the student's first assessment score with the student's engagement in that same course. The course activities include *oucontent*, *forumng*, *dataplus*, *URL*, *homepage,oucollaborate, resource, subpage, and glossary*.

**Figure 9:**
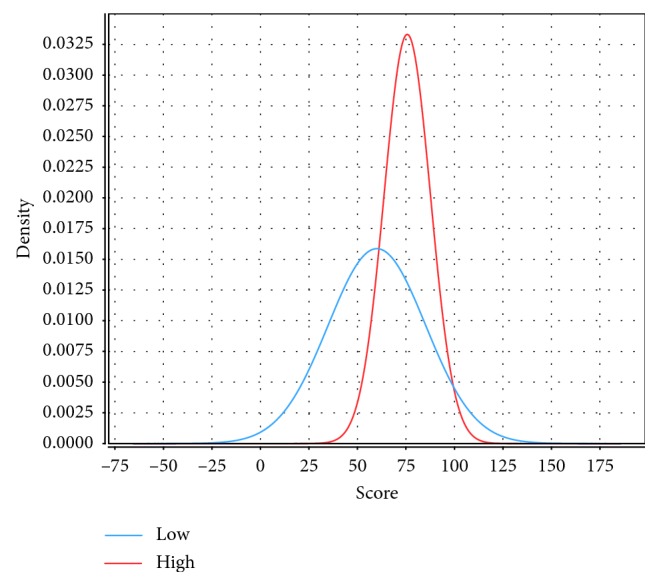
Visualization of the predicted engagement level of a student and the score of that student on the assessment based on the testing data.

As shown in Figure 9, we plotted the score on the first assessment and the predicted student engagement for the test data. The graph shows that high-engagement students usually earn better assessment scores in OU courses; this result may be due to their increased access of course content and increased participation in discussion forums. In contrast, low-engagement students tend to have lower scores on the first assessment; this result may be due to poor exam preparation, poor time management, limited access of course content, and low participation in discussion forums.

Furthermore, the results also indicate that a student's participation in VLE activities (e.g., discussion forums and course content access) positively affects student engagement and the student's scores in the course.

### 5.3. Development of an Engagement Prediction System

**Figure 10:**
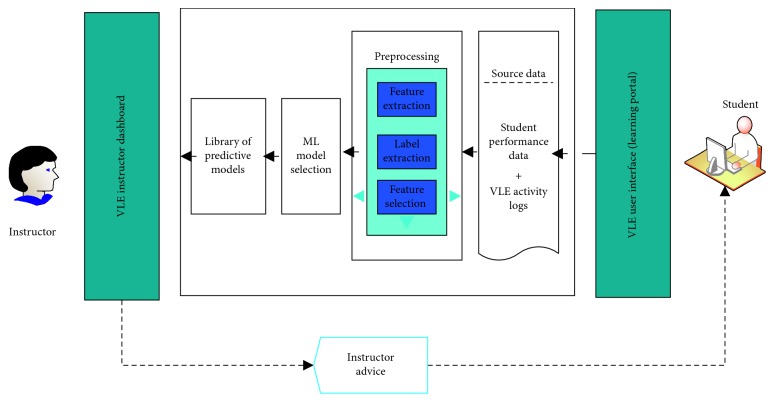
Structure of the student engagement prediction system.

We designed an engagement prediction system based on the results of the current study.  Figure 10 demonstrates the interaction between the student engagement prediction system and the VLE. The main components of the proposed student engagement prediction system are detailed as follows:

#### 5.3.1. Learning Portal (VLE)

The VLE is a web-based system that offers students a variety of functions such as enrolling in courses, solving problems, completing assessments, downloading materials, and performing activities. The students can interact with the VLE daily to complete the course assessments for the classes in which they are enrolled.

#### 5.3.2. Source Data

When students interact with the VLE system to complete a course assessment, their activities are recorded in the log file, and the student performance data are recorded in the student database.

#### 5.3.3. Preprocessing

The preprocessing module extracts input-related features and engagement labels from the student log data and transforms those data into a format acceptable for input into ML algorithms.

#### 5.3.4. ML Model Selection

Based on the ML performances for the student log data, this module selects the best ML model for making student engagement predictions.

#### 5.3.5. Library of Predictive Models

This module is responsible for predicting student engagement based on unknown student data or predicting low-engagement students in courses using the best ML classifier (J48, DT, JRIP, and GBT).

#### 5.3.6. Instructor Dashboard

Because the decision tree rules are in the form of if/then rules, it is difficult for the instructor to understand these rules. Consequently, the instructor dashboard is a computer program that interprets these rules and displays them in the form of a graph to provide valuable information about student engagement in VLE activities to the instructor. Moreover, the instructor can predict individual student engagement and then send pertinent intervention advice to low-engagement students.

### 5.4. OU Analysis Dashboard for the Current Study

**Figure 11:**
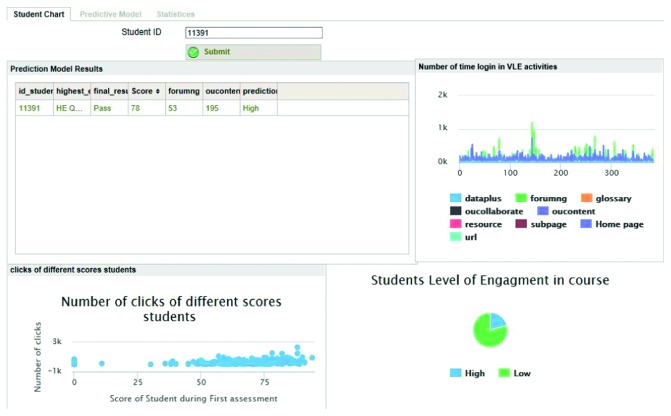
Snapshot of the current study dashboard.

Although the above experiments provide an overview of the performance of predictive models, they do not provide real-time information for the instructor. Therefore, to provide real-time information to the instructor, a prototype of an online dashboard for the OU was designed and is presented in Figure 11. The concept of the dashboard is described as follows:

The proposed instructor dashboard has two main components: a prediction part and a data visualization part. In the visualization part, an activity chart shows the number of times a student has logged in to each activity or to the VLE through the first assessment. The other part shows individual student engagement in each assessment.

After the model is developed, it can be applied to real student data; subsequently, at any moment in time, the model shows a student's engagement level for different assessments, materials, and activities. In other words, the model shows the number of times a student's uses VLE activities, such as *forumng* or *oucontent*.

The dashboard allows course instructors without IT skills to acquire up-to-date predictions about student engagement for each assessment and each activity and to make accurate decisions about students to reduce the student dropout rate. Additionally, the instructor can determine the reason for a student's low-engagement level.

A teacher can use this dashboard to contact students who have not accessed the course materials and who have not participated in VLE activities. The predictive model also generates a list of students who have low-engagement during the assessment.

Moreover, the predictive portion of the dashboard provides some statistical information about students and their course assignments. For example, first, the predictive portion identifies the top five most popular activities during the first assessment of the course; second, it finds the percentage of low- and high-level engagement students for the current course assessment.

Furthermore, a graphical representation of student interactions with course activities allows instructors to evaluate a student's behavior in a few seconds and to give feedback in real time.

This visualization allows the instructor to assess the effect of redesigning the course or material. Furthermore, the visualization enables the teaching staff to receive feedback on their teaching practices.

### 5.5. Predictive Model Application in a Web-Based System

A teacher can use the predictive model to receive the following feedback on a lesson or assignment and then deliver a response through the e-learning system.

1. When student engagement in particular activities or materials decreases, it may be useful for the instructor to ask why a majority of the students are not accessing the relevant activities or materials. One possible cause is that the materials delivered in the course are ineffective. Accordingly, the instructor should improve the quality of the instructional materials to satisfy students because satisfied students are more innovative, put more effort into their studies, and will recommend the course other students.
2. When a student engages less in the discussion forum or OU content or never accesses the OU content, the instructor can e-mail (disengagement trigger) the student to determine what materials the student is having difficulty with and to determine why the student is contributing less to the course. The instructor can also offer advice about the course. This advice may help to increase the student's awareness of their productive or unproductive behavior, which might increase their level of engagement.
3. The instructor can predict individual student difficulties for each assessment and recommend relevant materials and activities to students for the next assessment.
4. Instructor contributions to the discussion forum can also increase student engagement.
5. This model can help the instructor find the most important activities for students, increase student engagement, and help students achieve high scores on course assessments.
6. The teacher can use the model to increase student engagement. When the student engagement level in a course is low, the instructor can redesign the course, to improve the student interactions within the course.

This tool can help instructors to design materials such that students will remain engaged during the course assessment.

## 6. Conclusions and Future Work

Predicting low-engagement students is important in e-learning systems because it allows teachers to understand the behavior of students for different course activities. We collected data from an e-learning system (VLE) and formatted these data in a form suitable for input into an ML. We then performed various experiments based on these data. We applied several ML classification algorithms to our data and evaluated them using cross-validation techniques.

The results of the first experiment showed that *DT*, *J48*, *JRIP*, and *GBT* are the most appropriate algorithms for predicting low-engagement students during an OU assessment. Table 8 and Figure 5 reveal that the true-positive rate (recall) of the J48 model is slightly higher than the alternatives and that the J48 model successfully identifies the students who truly exhibit low-engagement during assessment activities.

The results of the second experiment indicate that the most important variables for predicting low-engagement in an OU assessment are clicks on *oucontent*, *forumng*, *subpage,* and *homepage*.

Because the current study used only student activity data from the OU system, the analysis focused on whether these data could be used to predict low-engagement students based on the assessment results. Student engagement is a complex problem that also depends on factors such as teaching experience, course design, teaching style, and course concepts. These factors must be further investigated in the context of student engagement.

In future work, we plan to evaluate the total number of students' clicks for each assessment, course design, teaching experience, and teaching style in an OU course and then use collaborative filtering to recommend materials and lectures for low-engagement students. This approach will help students achieve higher grades on the final exam.

## Figures and Tables

**Algorithm 1:**
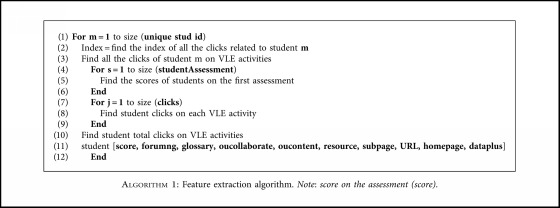
Feature extraction algorithm. *Note*: *score on the assessment (score)*.

**Table 1:** Correlation analysis and descriptive statistics for the activities and the students' level of engagement.

Variables	*r*	*P*	Std. deviation	Mean
Dataplus	0.064	0.383	0.58475	0.0940
Forumng	0.464^*∗∗*^	0.000	141.59343	91.3864
Glossary	−0.050	0.325	0.84851	0.1018
Oucollaborate	0.182^*∗∗*^	0.000	1.33278	0.5561
Oucontent	0.482^*∗∗*^	0.000	86.63507	116.1645
Resource	0.124^*∗*^	0.015	3.06710	1.7990
Subpage	0.423^*∗∗*^	0.000	16.57542	21.14.10
Homepage	0.542^*∗∗*^	0.000	57.60067	55.4961
URL	0.376^*∗∗*^	0.000	9.26278	7.3055
Score on the assessment	0.351^*∗∗*^	0.000	20.74514	65.7285

*Note*. An asterisk indicates a correlation significant at the 0.05 level (2-tailed). Features with large correlation coefficients (*P*>0.05) do not have an asterisk. Features significant at the *P*>0.05 level are indicated by an asterisk, and features significant at the *P*>0.01 level are indicated by double asterisks.

**Table 2:** Confusion matrix of the decision tree (DT) isolating two classes of students' engagement predictions.

Actual	Predicted		
	True low	True high	Class precision
Pred. low	TP = 283	FN = 20	93.40%
Pred. high	FP = 34	TN = 46	57.50%
Class recall	89.27%	69.70%	

**Table 3:** Confusion matrix of the J48 model when predicting two classes of students' engagement.

Actual	Predicted		
	True low	True high	Class precision
Pred. low	TP = 289	FN = 16	94.75%
Pred. high	FP = 28	TN = 50	64.10%
Class recall	91.17%	75.76%	

**Table 4:** Confusion matrix of the JRIP model when predicting two classes of students' engagement.

Actual	Predicted		
	True low	True high	Class precision
Pred. low	TP = 227	FN = 16	93.42%
Pred. high	FP = 48	TN = 92	65.71%
Class recall	82.55%	85.19%	

**Table 5:** Confusion matrix of the gradient-boosted tree (GBT) model when predicting two classes of students' engagement.

Actual	Predicted		
	True low	True high	Class precision
Pred. low	TP = 294	FN = 29	91.02%
Pred. high	FP = 23	TN = 37	61.67%
Class recall	92.74%	56.06%	

**Table 6:** Confusion matrix of the classification and regression tree (CART) model when predicting two classes of students' engagement.

Actual	Predicted		
	True low	True high	Class precision
Pred. low	TP = 235	FN = 28	89.35%
Pred. high	FP = 40	TN = 80	66.67%
Class recall	85.45%	74.07%	

**Table 7:** Confusion matrix of the Naive Bayes (Kernel) classifier when predicting two classes of students' engagement.

Actual	Predicted		
	True low	True high	Class precision
Pred. low	TP = 282	FN = 31	90.10%
Pred. high	FP = 34	TN = 34	50.00%
Class recall	89.24%	52.31%	

**Table 8:** Accuracy, kappa, and recall of the ML models used in the current study.

Model	Accuracy (%)	Kappa	Recall
Decisions tree	85.91	0.534	0.934
JRIP	83.27	0.621	0.934
J48	88.52	0.630	0.947
Gradient-boosted trees	86.43	0.503	0.910
CART	82.25	0.572	0.893
Naïve Bayes	82.93	0.408	0.900

## Data Availability

The current study data are publicly available online (https://analyse.kmi.open.ac.uk/open_dataset) for research purposes. Ethical clearance was granted by the Open University, UK. No participants' personal information (e.g., name or address) was included in this study.

## References

[1] Shishehchi S., Banihashem S. Y., Zin N. A. M., Noah S. A. M. Review of personalized recommendation techniques for learners in e-learning systems.

[2] Olazabalaga M. I., Garrido C. C., Ruiz G. U. (2016). Research on MOOCs: trends and methodologies. *Porta Linguarum*.

[3] Ye C., Biswas G. (2014). Early prediction of student dropout and performance in MOOCs using higher granularity temporal information. *Journal of Learning Analytics*.

[4] Mutahi J., Kinai A., Bore N., Diriye A., Weldemariam K. Studying engagement and performance with learning technology in an African classroom.

[5] Simpson O. 22% - can we do better?’ The CWP Retention Literature Review.

[6] Kuzilek J., Hlosta M., Herrmannova D., Zdrahal Z., Wolff A. OU analyse: analysing at-risk students at the Open University.

[7] Jung Y., Lee J. (2018). Learning engagement and persistence in massive open online courses (MOOCS). *Computers & Education*.

[8] Kushwaha R. C., Singhal A., Chaurasia P. K. (2015). Study of students’ performance in learning management system. *International Journal of Contemporary Research in Computer Science and Technology (IJCRCST)*.

[9] Holmes N. (2018). Engaging with assessment: increasing student engagement through continuous assessment. *Active Learning in Higher Education*.

[10] Blin F., Munro M. (2008). Why hasn’t technology disrupted academics’ teaching practices? Understanding resistance to change through the lens of activity theory. *Computers & Education*.

[11] Herrmannova D., Hlosta M., Kuzilek J., Zdrahal Z. Evaluating weekly predictions of at-risk students at the open university: results and issues.

[12] Mogus A. M., Djurdjevic I., Suvak N. (2012). The impact of student activity in a virtual learning environment on their final mark. *Active Learning in Higher Education*.

[13] Hlosta M., Zdrahal Z., Zendulka J. Ouroboros: early identification of at-risk students without models based on legacy data.

[14] Coates H. (2006). *Student Engagement in Campus-Based and Online Education*.

[15] Staikopoulos A., O’Keeffe I., Yousuf B. Enhancing student engagement through personalized motivations.

[16] Wang Y., Baker R. (2015). Content or platform: why do students complete MOOCs?. *Journal of Online Learning and Teaching*.

[17] Stovall Engagement and online learning. UIS community of practice for e-learning. http://otel.uis.edu/copel/EngagementandOnlineLearning.ppt.

[18] Beer C. Online student engagement: new measures for new methods.

[19] Douglas I., Alemanne N. D. Measuring student participation and effort.

[20] Bulger M. E., Mayer R. E., Almeroth K. C., Blau S. D. (2008). Measuring learner engagement in computer-equipped college classrooms. *Journal of Educational Multimedia and Hypermedia*.

[21] Tempelaar D. T., Rienties B., Giesbers B. (2015). In search for the most informative data for feedback generation: learning analytics in a data-rich context. *Computers in Human Behaviour*.

[22] Corrigan O., Smeaton A. F., Glynn M., Smyth S. Using educational analytics to improve test performance.

[23] Jayaprakash S. M., Moody E. W., Lauría E. J. M., Regan J. R., Baron J. D. (2014). Early alert of academically at-risk students: an open source analytics initiative. *Journal of Learning Analytics*.

[24] Holland J. H. (1992). *Adaptation in Natural and Artificial Systems: An Introductory Analysis with Applications to Biology, Control and Artificial Intelligence*.

[25] Greller W., Ebner M., Schön M., Kalz M., Ras E. (2014). Learning analytics: from theory to practice–data support for learning and teaching. *Computer Assisted Assessment. Research into E-Assessment*.

[26] Leitner P., Khalil M., Ebner M., Peña-Ayala A. (2017). Learning analytics in higher education—a literature review. *Learning Analytics: Fundaments, Applications, and Trends: A View of the Current State of the Art to Enhance E-Learning*.

[27] Wolff A., Zdrahal Z., Herrmannova D., Knoth P., Peña-Ayala A. (2013). Predicting student performance from combined data sources. *Educational Data Mining. Studies in Computational Intelligence*.

[28] Kotsiantis S., Pierrakeas C., Pintelas P. (2004). Predicting students’ performance in distance learning using machine learning techniques. *Applied Artificial Intelligence*.

[29] Arnold K. E., Pistilli M. D. Course signals at Purdue: using learning analytics to increase student success.

[30] Kai S., Andres J. M. L., Paquette L. Predicting student retention from behaviour in an online orientation course.

[31] Edelstein H. A. (1999). *Introduction to Data Mining and Knowledge Discovery*.

[32] Krause K. L., Coates H. (2008). Students’ engagement in first-year university. *Assessment & Evaluation in Higher Education*.

[33] Zhuang M., Demartini G., Toms E. G. Understanding engagement through search behaviour.

[34] Hlosta M., Herrmannova D., Vachova L. Modelling student online behaviour in a virtual learning environment.

[35] Mitchell T., Buchanan B., De Jong G., Dietterich T., Rosenbloom P., Waibel A. (1989). Machine learning. *Annual Review of Computer Science*.

[36] Wolff A., Zdrahal Z., Herrmannova D., Kuzilek J., Hlosta M. Developing predictive models for early detection of at-risk students on distance learning modules.

[37] Guo P. J., Kim J., Rubin R. How video production affects student engagement: an empirical study of MOOC videos.

[38] Bonafini F. C., Chae C., Park E., Jablokow K. W. (2017). How much does student engagement with videos and forums in a MOOC affect their achievement?. *Online Learning*.

[39] Ramesh A., Goldwasser D., Huang B., Daum H., Getoor L. Learning latent engagement patterns of students in online courses.

[40] Ramesh A., Goldwasser D., Huang B., Daumé H., Getoor L. Modeling learner engagement in MOOCs using probabilistic soft logic.

[41] Manwaring K. C., Larsen R., Graham C. R., Henrie C. R., Halverson L. R. (2017). Investigating student engagement in blended learning settings using experience sampling and structural equation modelling. *Internet and Higher Education*.

[42] Aguiar E., Ambrose G. A., Chawla N. V., Goodrich V., Brockman J. (2014). Engagement vs performance: using electronic portfolios to predict first semester engineering student persistence. *Journal of Learning Analytics*.

[43] Thomas C., Jayagopi D. B. Predicting student engagement in classrooms using facial behavioral cues.

[44] Atherton M., Shah M., Vazquez J., Griffiths Z., Jackson B., Burgess C. (2017). Using learning analytics to assess student engagement and academic outcomes in open access enabling programmes. *Journal of Open, Distance and e-Learning*.

[45] Bosch N. Detecting student engagement: human versus machine.

[46] Kizilcec R. F., Piech C., Schneider E. Deconstructing disengagement: analyzing learner subpopulations in massive open online courses.

[47] Milligan C., Littlejohn A., Margaryan A. (2013). Patterns of engagement in connectivist MOOCs. *Journal of Online Learning and Teaching*.

[48] Ding L., Er E., Orey M. (2018). An exploratory study of student engagement in gamified online discussions. *Computers & Education*.

[49] Wells M., Wollenschlaeger A., Lefevre D., Magoulas G. D., Poulovassilis A. Analysing engagement in an online management programme and implications for course design.

[50] Pardo A., Han F., Ellis R. A. Exploring the relation between self-regulation, online activities, and academic performance: a case study.

[51] Bahati B., Uno F., Tedre M. (2017). Can student engagement in online courses predict performance on online knowledge surveys?. *International Journal of Learning, Teaching and Educational Research*.

[52] Hamid S. S. A., Admodisastro N., Manshor N., Kamaruddin A., Ghani A. A. A., Ghazali R., Deris M., Nawi N., Abawajy J. (2018). Dyslexia adaptive learning model: student engagement prediction using machine learning approach. *Advances in Intelligent Systems and Computing*.

[53] Bote-Lorenzo M. L., Gomez-Sanchez E. Predicting the decrease of engagement indicators in a MOOC.

[54] Marks H. M. (2000). Student engagement in instructional activity: patterns in the elementary middle and high school years. *American Educational Research Journal*.

[55] Kuh G. D. (2001). Assessing what really matters to student learning inside the national survey of student engagement. *Change: The Magazine of Higher Learning*.

[56] Rodgers T. (2008). Student engagement in the e-learning process and the impact on their grade. *International Journal of Cyber Society and Education*.

[57] Kabra R., Bichkar R. (2011). Performance prediction of engineering students using decision trees. *International Journal of Computer Applications*.

[58] Sharma A. K., Sahni S. (2011). A comparative study of classification algorithms for spam email data analysis. *International Journal on Computer Science and Engineering*.

[59] Asif R., Merceron A., Pathan M. K. (2014). Predicting student academic performance at degree level: a case study. *International Journal of Intelligent Systems and Applications*.

[60] Wolff A., Zdrahal Z. Improving retention by identifying and supporting at-risk students.

[61] Rajput A., Aharwal R. P., Dubey M., Saxena S., Raghuvanshi M. (2011). J48 and JRIP rules for e-governance data. *International Journal of Computer Science and Security (IJCSS)*.

[62] Chapter 5, Classification Methods, March 2018, http://www.d.umn.edu/~padhy005/Chapter5.html

[63] Lakshmi T. M., Martin A., Begum R. M., Venkatesan V. P. (2013). An analysis on performance of decision tree algorithms using student’s qualitative data. *International Journal of Modern Education and Computer Science*.

[64] Márquez-Vera C., Cano A., Romero C., Noaman A. Y. M., Mousa Fardoun H., Ventura S. (2015). Early dropout prediction using data mining: a case study with high school students. *Expert Systems*.

[65] Cohen W. W. Fast effective rule induction.

[66] O’Mahony M. P., Cunningham P., Smyth B. An assessment of machine learning techniques for review recommendation.

[67] Mousa H., Maghari A. (2017). School student’s performance prediction using data mining classification. *International Journal of Advance Research in Computer and Communication Technology*.

[68] Kuzilek J., Hlosta M., Zdrahal Z. (2017). Open university learning analytics dataset. *Scientific Data*.

[69] Parsons S. A., Nuland L. R., Parsons A. W. (2014). The ABCs of student engagement. *Phi Delta Kappan*.

[70] Siemens G. (2004). Connectivism: a learning theory for the digital age. *International Journal of Instructional Technology and Distance Learning*.

[71] Prince M. (2004). Does active learning work? A review of the research. *Journal of Engineering Education*.

[72] Vrasidas C., McIsaac M. S. (1999). Factors influencing interaction in an online course. *American Journal of Distance Education*.

[73] Albuquerque R. M. D., Bezerra A. A., Souza D. A. D. Using neural networks to predict the future performance of students.

[74] Cawley G. C., Talbot N. L. C. (2006). Gene selection in cancer classification using sparse logistic regression with Bayesian regularization. *Bioinformatics*.

[75] Rovira S., Puertas E., Igual L. (2017). Data-driven system to predict academic grades and dropout. *PLoS One*.

[76] Hussain M., Zhu W., Zhang W., Abidi S. M. R., Ali S. (2018). Using machine learning to predict student difficulties from learning session data. *Artificial Intelligence Review*.

[77] Freitas A. A. (2002). *Data Mining and Knowledge Discovery with Evolutionary Algorithms*.

[78] Villagrá-Arnedo C., Gallego-Duraìn F. J., Compan˜-Rosique P., Llorens-Largo F., Molina-Carmona R. (2016). Predicting academic performance from behavioural and learning data. *International Journal of Design & Nature and Ecodynamics*.

[79] Brace N., Kemp R., Snelgar R. (2006). *SPSS for Psychologists: A Guide to Data Analysis Using SPSS for Windows, Version 12 and 13*.

[80] Zacharis N. Z. (2015). A multivariate approach to predicting student outcomes in web-enabled blended learning courses. *Internet and Higher Education*.

[81] Luan J., Zhao C.-M. (2006). Practicing data mining for enrolment management and beyond. *New Directions for Institutional Research*.

[82] Kovacic Z. Early prediction of student success: mining students’ enrolment data.

[83] Wolff A., Zdrahal Z., Nikolov A., Pantucek M. Improving retention: predicting at-risk students by analysing clicking behaviour in a virtual learning environment.

[84] Jung I., Choi S., Lim C., Leem J. (2002). Effects of different types of interaction on learning achievement, satisfaction and participation in web-based instruction. *Innovations in Education and Teaching International*.

[85] Roberts J., Styron R. (2010). Student satisfaction and persistence: factors vital to student retention. *Research in Higher Education Journal*.

[86] Breiman L., Friedman J. H., Olshen R. A., Stone C. J. (1984). *Classification and Regression Trees*.

[87] Romero C., López M.-I., Luna J.-M., Ventura S. (2013). Predicting students’ final performance from participation in on-line discussion forums. *Computers & Education*.

[88] Sathyadevan S., Nair R. R. Comparative analysis of decision tree algorithms: ID_3_, C_4.5_ and random forest.

[89] Skinner E. A., Pitzer J. R., Christenson S. L., Reschly A. L., Wylie C. (2012). Developmental dynamics of student engagement, coping, and everyday resilience. *Handbook of Research on Student Engagement*.

